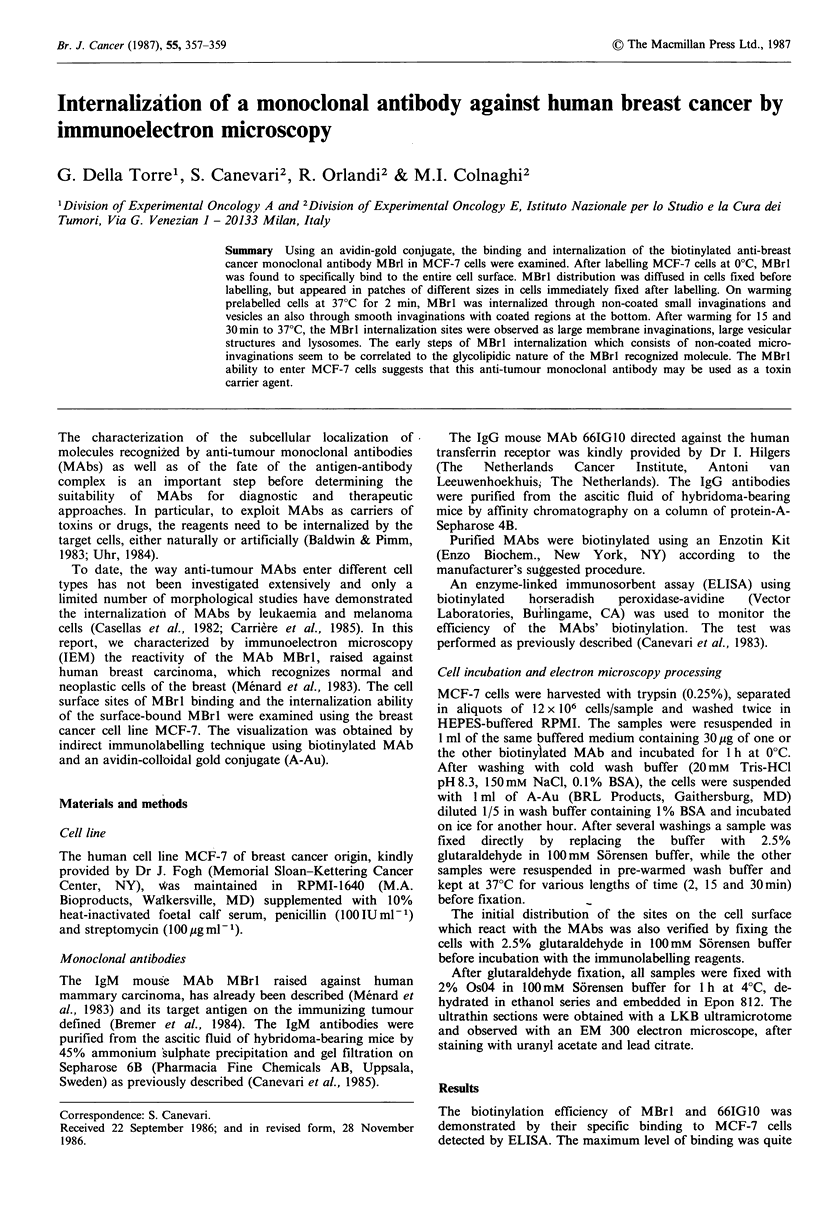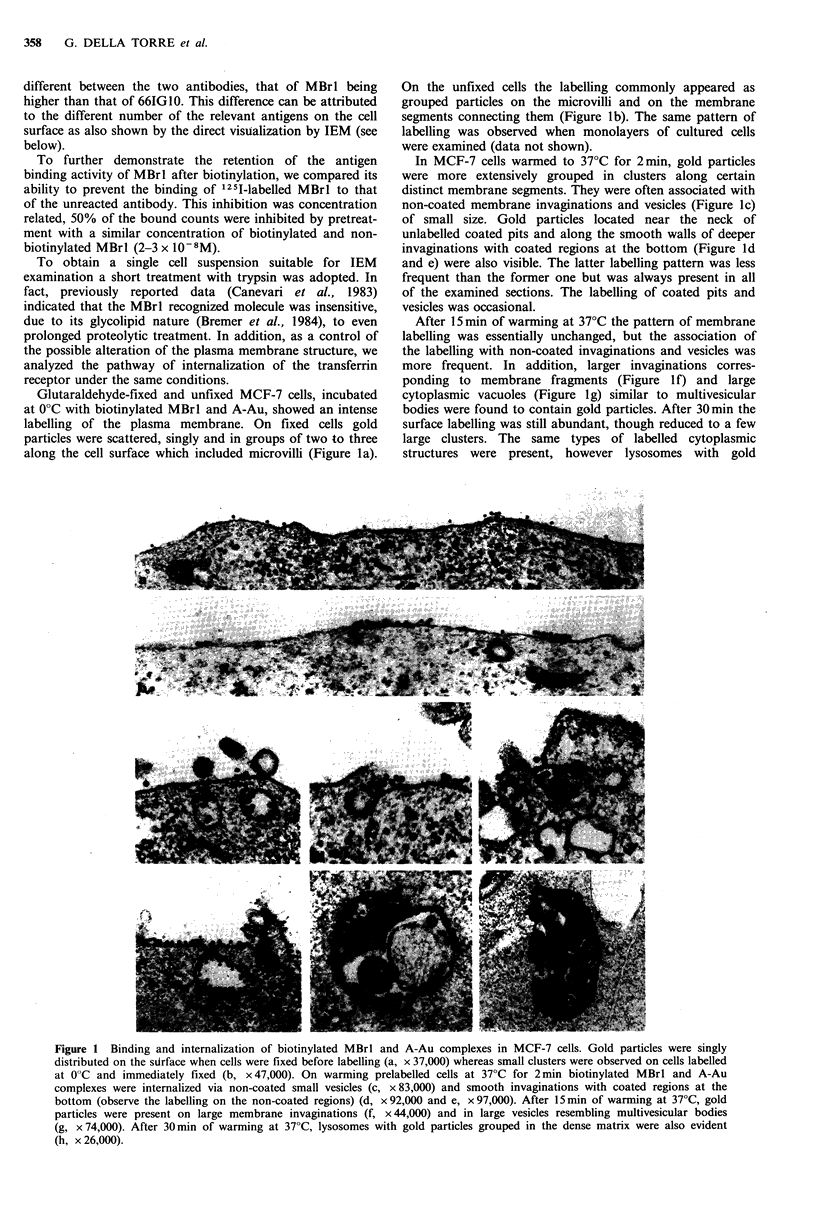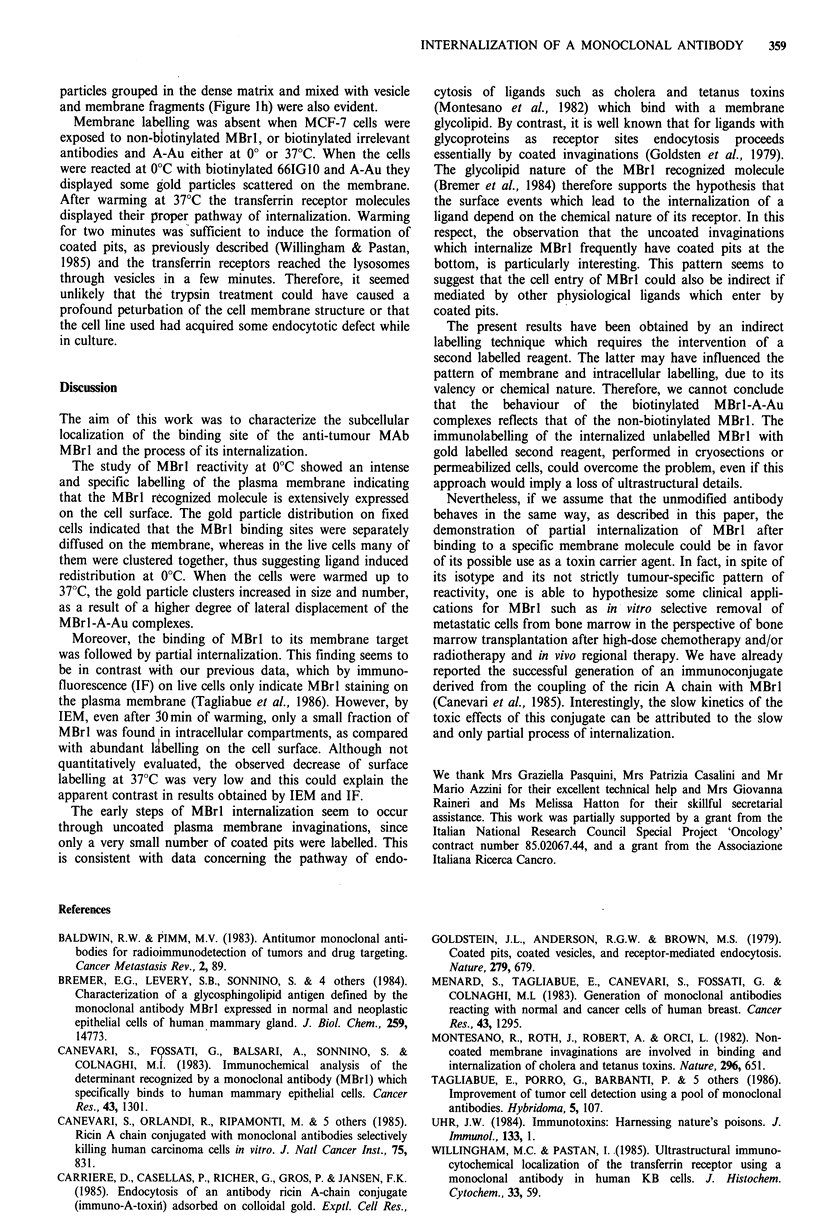# Internalization of a monoclonal antibody against human breast cancer by immunoelectron microscopy.

**DOI:** 10.1038/bjc.1987.72

**Published:** 1987-04

**Authors:** G. Della Torre, S. Canevari, R. Orlandi, M. I. Colnaghi

## Abstract

**Images:**


Br. J. Cancer (1987), 55, 357–359 © The Macmillan Press Ltd., 1987

### Internalization of a monoclonal antibody against human breast cancer by immunoelectron microscopy

G. Della Torre[1], S. Canevari[2], R. Orlandi[2] & M.I. Colnaghi[2]

[1]*Division of Experimental Oncology A and* [2]*Division of Experimental Oncology E, Istituto Nazionale per lo Studio e la Cura dei Tumori, Via G. Venezian 1 – 20133 Milan, Italy*

**Summary** Using an avidin-gold conjugate, the binding and internalization of the biotinylated anti-breast cancer monoclonal antibody MBrl in MCF-7 cells were examined. After labelling MCF-7 cells at 0°C, MBrl was found to specifically bind to the entire cell surface. MBrl distribution was diffused in cells fixed before labelling, but appeared in patches of different sizes in cells immediately fixed after labelling. On warming prelabelled cells at 37°C for 2 min, MBrl was internalized through non-coated small invaginations and vesicles an also through smooth invaginations with coated regions at the bottom. After warming for 15 and 30 min to 37°C, the MBrl internalization sites were observed as large membrane invaginations, large vesicular structures and lysosomes. The early steps of MBrl internalization which consists of non-coated micro-invaginations seem to be correlated to the glycolipidic nature of the MBrl recognized molecule. The MBrl ability to enter MCF-7 cells suggests that this anti-tumour monoclonal antibody may be used as a toxin carrier agent.

The characterization of the subcellular localization of molecules recognized by anti-tumour monoclonal antibodies (MAbs) as well as of the fate of the antigen-antibody complex is an important step before determining the suitability of MAbs for diagnostic and therapeutic approaches. In particular, to exploit MAbs as carriers of toxins or drugs, the reagents need to be internalized by the target cells, either naturally or artificially (Baldwin & Pimm, 1983; Uhr, 1984).

To date, the way anti-tumour MAbs enter different cell types has not been investigated extensively and only a limited number of morphological studies have demonstrated the internalization of MAbs by leukaemia and melanoma cells (Casellas *et al.*, 1982; Carrière *et al.*, 1985). In this report, we characterized by immunoelectron microscopy (IEM) the reactivity of the MAb MBrl, raised against human breast carcinoma, which recognizes normal and neoplastic cells of the breast (Ménard *et al.*, 1983). The cell surface sites of MBrl binding and the internalization ability of the surface-bound MBrl were examined using the breast cancer cell line MCF-7. The visualization was obtained by indirect immunolabelling technique using biotinylated MAb and an avidin-colloidal gold conjugate (A-Au).

### Materials and methods

### *Cell line*

The human cell line MCF-7 of breast cancer origin, kindly provided by Dr J. Fogh (Memorial Sloan–Kettering Cancer Center, NY), was maintained in RPMI-1640 (M.A. Bioproducts, Walkersville, MD) supplemented with 10% heat-inactivated foetal calf serum, penicillin ($100 IU ml^{-1}$) and streptomycin ($100 \mu g ml^{-1}$).

### *Monoclonal antibodies*

The IgM mouse MAb MBrl raised against human mammary carcinoma, has already been described (Ménard *et al.*, 1983) and its target antigen on the immunizing tumour defined (Bremer *et al.*, 1984). The IgM antibodies were purified from the ascitic fluid of hybridoma-bearing mice by 45% ammonium sulphate precipitation and gel filtration on Sepharose 6B (Pharmacia Fine Chemicals AB, Uppsala, Sweden) as previously described (Canevari *et al.*, 1985).

The IgG mouse MAb 66IG10 directed against the human transferrin receptor was kindly provided by Dr I. Hilgers (The Netherlands Cancer Institute, Antoni van Leeuwenhoekhuis, The Netherlands). The IgG antibodies were purified from the ascitic fluid of hybridoma-bearing mice by affinity chromatography on a column of protein-A-Sepharose 4B.

Purified MAbs were biotinylated using an Enzotin Kit (Enzo Biochem., New York, NY) according to the manufacturer's suggested procedure.

An enzyme-linked immunosorbent assay (ELISA) using biotinylated horseradish peroxidase-avidine (Vector Laboratories, Burlingame, CA) was used to monitor the efficiency of the MAbs' biotinylation. The test was performed as previously described (Canevari *et al.*, 1983).

### *Cell incubation and electron microscopy processing*

MCF-7 cells were harvested with trypsin (0.25%), separated in aliquots of $12 \times 10^6$ cells/sample and washed twice in HEPES-buffered RPMI. The samples were resuspended in 1 ml of the same buffered medium containing $30 \mu g$ of one or the other biotinylated MAb and incubated for 1 h at 0°C. After washing with cold wash buffer (20 mM Tris-HCl pH 8.3, 150 mM NaCl, 0.1% BSA), the cells were suspended with 1 ml of A-Au (BRL Products, Gaithersburg, MD) diluted 1/5 in wash buffer containing 1% BSA and incubated on ice for another hour. After several washings a sample was fixed directly by replacing the buffer with 2.5% glutaraldehyde in 100 mM Sörensen buffer, while the other samples were resuspended in pre-warmed wash buffer and kept at 37°C for various lengths of time (2, 15 and 30 min) before fixation.

The initial distribution of the sites on the cell surface which react with the MAbs was also verified by fixing the cells with 2.5% glutaraldehyde in 100 mM Sörensen buffer before incubation with the immunolabelling reagents.

After glutaraldehyde fixation, all samples were fixed with 2% Os04 in 100 mM Sörensen buffer for 1 h at 4°C, dehydrated in ethanol series and embedded in Epon 812. The ultrathin sections were obtained with a LKB ultramicrotome and observed with an EM 300 electron microscope, after staining with uranyl acetate and lead citrate.

### Results

The biotinylation efficiency of MBrl and 66IG10 was demonstrated by their specific binding to MCF-7 cells detected by ELISA. The maximum level of binding was quite

Correspondence: S. Canevari.
Received 22 September 1986; and in revised form, 28 November 1986.

different between the two antibodies, that of MBr1 being higher than that of 66IG10. This difference can be attributed to the different number of the relevant antigens on the cell surface as also shown by the direct visualization by IEM (see below).

To further demonstrate the retention of the antigen binding activity of MBr1 after biotinylation, we compared its ability to prevent the binding of $^{125}$I-labelled MBr1 to that of the unreacted antibody. This inhibition was concentration related, 50% of the bound counts were inhibited by pretreatment with a similar concentration of biotinylated and non-biotinylated MBr1 ($2–3 \times 10^{-8}$M).

To obtain a single cell suspension suitable for IEM examination a short treatment with trypsin was adopted. In fact, previously reported data (Canevari *et al.,* 1983) indicated that the MBr1 recognized molecule was insensitive, due to its glycolipid nature (Bremer *et al.,* 1984), to even prolonged proteolytic treatment. In addition, as a control of the possible alteration of the plasma membrane structure, we analyzed the pathway of internalization of the transferrin receptor under the same conditions.

Glutaraldehyde-fixed and unfixed MCF-7 cells, incubated at 0°C with biotinylated MBr1 and A-Au, showed an intense labelling of the plasma membrane. On fixed cells gold particles were scattered, singly and in groups of two to three along the cell surface which included microvilli (Figure 1a). On the unfixed cells the labelling commonly appeared as grouped particles on the microvilli and on the membrane segments connecting them (Figure 1b). The same pattern of labelling was observed when monolayers of cultured cells were examined (data not shown).

In MCF-7 cells warmed to 37°C for 2 min, gold particles were more extensively grouped in clusters along certain distinct membrane segments. They were often associated with non-coated membrane invaginations and vesicles (Figure 1c) of small size. Gold particles located near the neck of unlabelled coated pits and along the smooth walls of deeper invaginations with coated regions at the bottom (Figure 1d and e) were also visible. The latter labelling pattern was less frequent than the former one but was always present in all of the examined sections. The labelling of coated pits and vesicles was occasional.

After 15 min of warming at 37°C the pattern of membrane labelling was essentially unchanged, but the association of the labelling with non-coated invaginations and vesicles was more frequent. In addition, larger invaginations corresponding to membrane fragments (Figure 1f) and large cytoplasmic vacuoles (Figure 1g) similar to multivesicular bodies were found to contain gold particles. After 30 min the surface labelling was still abundant, though reduced to a few large clusters. The same types of labelled cytoplasmic structures were present, however lysosomes with gold

**Figure 1** Binding and internalization of biotinylated MBr1 and A-Au complexes in MCF-7 cells. Gold particles were singly distributed on the surface when cells were fixed before labelling (a, ×37,000) whereas small clusters were observed on cells labelled at 0°C and immediately fixed (b, ×47,000). On warming prelabelled cells at 37°C for 2 min biotinylated MBr1 and A-Au complexes were internalized via non-coated small vesicles (c, ×83,000) and smooth invaginations with coated regions at the bottom (observe the labelling on the non-coated regions) (d, ×92,000 and e, ×97,000). After 15 min of warming at 37°C, gold particles were present on large membrane invaginations (f, ×44,000) and in large vesicles resembling multivesicular bodies (g, ×74,000). After 30 min of warming at 37°C, lysosomes with gold particles grouped in the dense matrix were also evident (h, ×26,000).

particles grouped in the dense matrix and mixed with vesicle and membrane fragments (Figure 1h) were also evident.

Membrane labelling was absent when MCF-7 cells were exposed to non-biotinylated MBr1, or biotinylated irrelevant antibodies and A-Au either at 0° or 37°C. When the cells were reacted at 0°C with biotinylated 66IG10 and A-Au they displayed some gold particles scattered on the membrane. After warming at 37°C the transferrin receptor molecules displayed their proper pathway of internalization. Warming for two minutes was sufficient to induce the formation of coated pits, as previously described (Willingham & Pastan, 1985) and the transferrin receptors reached the lysosomes through vesicles in a few minutes. Therefore, it seemed unlikely that the trypsin treatment could have caused a profound peturbation of the cell membrane structure or that the cell line used had acquired some endocytotic defect while in culture.

### Discussion

The aim of this work was to characterize the subcellular localization of the binding site of the anti-tumour MAb MBr1 and the process of its internalization.

The study of MBr1 reactivity at 0°C showed an intense and specific labelling of the plasma membrane indicating that the MBr1 recognized molecule is extensively expressed on the cell surface. The gold particle distribution on fixed cells indicated that the MBr1 binding sites were separately diffused on the membrane, whereas in the live cells many of them were clustered together, thus suggesting ligand induced redistribution at 0°C. When the cells were warmed up to 37°C, the gold particle clusters increased in size and number, as a result of a higher degree of lateral displacement of the MBr1-A-Au complexes.

Moreover, the binding of MBr1 to its membrane target was followed by partial internalization. This finding seems to be in contrast with our previous data, which by immunofluorescence (IF) on live cells only indicate MBr1 staining on the plasma membrane (Tagliabue *et al.*, 1986). However, by IEM, even after 30 min of warming, only a small fraction of MBr1 was found in intracellular compartments, as compared with abundant labelling on the cell surface. Although not quantitatively evaluated, the observed decrease of surface labelling at 37°C was very low and this could explain the apparent contrast in results obtained by IEM and IF.

The early steps of MBr1 internalization seem to occur through uncoated plasma membrane invaginations, since only a very small number of coated pits were labelled. This is consistent with data concerning the pathway of endocytosis of ligands such as cholera and tetanus toxins (Montesano *et al.*, 1982) which bind with a membrane glycolipid. By contrast, it is well known that for ligands with glycoproteins as receptor sites endocytosis proceeds essentially by coated invaginations (Goldsten *et al.*, 1979). The glycolipid nature of the MBr1 recognized molecule (Bremer *et al.*, 1984) therefore supports the hypothesis that the surface events which lead to the internalization of a ligand depend on the chemical nature of its receptor. In this respect, the observation that the uncoated invaginations which internalize MBr1 frequently have coated pits at the bottom, is particularly interesting. This pattern seems to suggest that the cell entry of MBr1 could also be indirect if mediated by other physiological ligands which enter by coated pits.

The present results have been obtained by an indirect labelling technique which requires the intervention of a second labelled reagent. The latter may have influenced the pattern of membrane and intracellular labelling, due to its valency or chemical nature. Therefore, we cannot conclude that the behaviour of the biotinylated MBr1-A-Au complexes reflects that of the non-biotinylated MBr1. The immunolabelling of the internalized unlabelled MBr1 with gold labelled second reagent, performed in cryosections or permeabilized cells, could overcome the problem, even if this approach would imply a loss of ultrastructural details.

Nevertheless, if we assume that the unmodified antibody behaves in the same way, as described in this paper, the demonstration of partial internalization of MBr1 after binding to a specific membrane molecule could be in favor of its possible use as a toxin carrier agent. In fact, in spite of its isotype and its not strictly tumour-specific pattern of reactivity, one is able to hypothesize some clinical applications for MBr1 such as *in vitro* selective removal of metastatic cells from bone marrow in the perspective of bone marrow transplantation after high-dose chemotherapy and/or radiotherapy and *in vivo* regional therapy. We have already reported the successful generation of an immunoconjugate derived from the coupling of the ricin A chain with MBr1 (Canevari *et al.*, 1985). Interestingly, the slow kinetics of the toxic effects of this conjugate can be attributed to the slow and only partial process of internalization.

We thank Mrs Graziella Pasquini, Mrs Patrizia Casalini and Mr Mario Azzini for their excellent technical help and Mrs Giovanna Raineri and Ms Melissa Hatton for their skillful secretarial assistance. This work was partially supported by a grant from the Italian National Research Council Special Project 'Oncology' contract number 85.02067.44, and a grant from the Associazione Italiana Ricerca Cancro.

### References

BALDWIN, R.W. & PIMM, M.V. (1983). Antitumor monoclonal antibodies for radioimmunodetection of tumors and drug targeting. *Cancer Metastasis Rev.*, **2**, 89.

BREMER, E.G., LEVERY, S.B., SONNINO, S. & 4 others (1984). Characterization of a glycosphingolipid antigen defined by the monoclonal antibody MBr1 expressed in normal and neoplastic epithelial cells of human mammary gland. *J. Biol. Chem.*, **259**, 14773.

CANEVARI, S., FOSSATI, G., BALSARI, A., SONNINO, S. & COLNAGHI, M.I. (1983). Immunochemical analysis of the determinant recognized by a monoclonal antibody (MBr1) which specifically binds to human mammary epithelial cells. *Cancer Res.*, **43**, 1301.

CANEVARI, S., ORLANDI, R., RIPAMONTI, M. & 5 others (1985). Ricin A chain conjugated with monoclonal antibodies selectively killing human carcinoma cells *in vitro*. *J. Natl Cancer Inst.*, **75**, 831.

CARRIERE, D., CASELLAS, P., RICHER, G., GROS, P. & JANSEN, F.K. (1985). Endocytosis of an antibody ricin A-chain conjugate (immuno-A-toxin) adsorbed on colloidal gold. *Exptl. Cell Res.*,

GOLDSTEIN, J.L., ANDERSON, R.G.W. & BROWN, M.S. (1979). Coated pits, coated vesicles, and receptor-mediated endocytosis. *Nature*, **279**, 679.

MENARD, S., TAGLIABUE, E., CANEVARI, S., FOSSATI, G. & COLNAGHI, M.L (1983). Generation of monoclonal antibodies reacting with normal and cancer cells of human breast. *Cancer Res.*, **43**, 1295.

MONTESANO, R., ROTH, J., ROBERT, A. & ORCI, L. (1982). Noncoated membrane invaginations are involved in binding and internalization of cholera and tetanus toxins. *Nature*, **296**, 651.

TAGLIABUE, E., PORRO, G., BARBANTI, P. & 5 others (1986). Improvement of tumor cell detection using a pool of monoclonal antibodies. *Hybridoma*, **5**, 107.

UHR, J.W. (1984). Immunotoxins: Harnessing nature's poisons. *J. Immunol.*, **133**, 1.

WILLINGHAM, M.C. & PASTAN, I. (1985). Ultrastructural immunocytochemical localization of the transferrin receptor using a monoclonal antibody in human KB cells. *J. Histochem. Cytochem.*, **33**, 59.